# Community perspectives on the Zambian Farm Block Development Program and an evaluation of household income factors: Lessons from Kawambwa district

**DOI:** 10.1371/journal.pone.0356479

**Published:** 2026-08-28

**Authors:** Obed Chanda

**Affiliations:** Department of Agriculture, Ministry of Agriculture, Mansa, Zambia; University of Zambia School of Veterinary Medicine, ZAMBIA

## Abstract

Since 2002, the Farm Block Development Program was one of the flagship initiatives within Zambia’s agricultural policy. This program was designed to promote commercialization of rural agricultural lands, to contribute to national food security, economic growth, and improved rural livelihoods. However, host community concerns remained untold. This paper aimed at shedding light on those concerns, and the factors associated with household incomes. A qualitative evaluation of submissions from Luena Farm Block residents in Kawambwa district in Zambia, showed that reticent project reception; fears of displacement, job scarcity, environmental damage, and cultural erosion were major issues for the local community. Further, a quantitative evaluation of data from 5286 respondents, using Tobit models established that 95% of household incomes were below Zambia’s official minimum wage, and the rural average income. Censored Least Absolute Deviation regression revealed that the household income factors were education, household size, cultivated area, and land tenure security. Environment-level income factors were health facility presence, and water access. The conclusion urged a cautious approach to achieve sustainable farm block development. Policy recommendations were that community needs and interests should be mainstreamed in the Farm Block Development Program. Education and health facilities should be promoted to enhance literacy and productivity. Mechanization should be promoted to expand crop areas and production efficiency. Land tenure security should be facilitated to protect fixed investments. Safe water access should be promoted to support irrigation and public health.

## 1. Introduction

The persistence of extreme poverty and hunger in the 21st century, was a drawback to the achievement of global sustainable development. However, the severity of this specter varied across different regions of the world. The 2024 Global Hunger Index had shown that significant progress was made in reducing hunger in Asia and South America, while the situation remained stagnant in Sub-Sahara Africa (SSA). Swargiary [1] and Wudil et al. [2] attributed the lack of progress in SSA to conflict, low productivity, climate change, and weak economic infrastructure to support industry and intra-regional trade. The United Nations Sustainable Development Goals (UNSDGs) number 1 and 2 represented a global vision to end poverty and hunger respectively. However, practical measures and actions through several global agencies had proved insufficient for ending poverty, and its offshoots like hunger in SSA.

Efforts to combat this scourge within the SSA region lacked financial backing for the enforcement of local strategies [3]. For instance, the African Union’s Maputo and Malabo declarations under the Comprehensive Africa Agriculture Development Program had its member countries agree to allocate at least 10% of their public expenditure budgets to agricultural production [4]. However, such a measure was not likely to materialize in some SSA countries like Zambia, without leaving other sectors worse off. Still, respective countries needed to “think global while acting local”, in their efforts to domesticate global and regional commitments to end poverty and hunger. Zambia’s Farm Block Development Program (FBDP) was designed as a response to this kind of challenge. This paper sought to analyze an aspect of this program in Zambia that exposes host community views and needs.

Meanwhile, the hunger situation in Zambia was described as serious by the 2024 Global Hunger Index, whereby the country was ranked 115^th^ out of 127 countries. Although this ranking marked a progressive shift from an extremely alarming situation registered circa 2000 [5], enough room existed for Zambia to emerge as one of the regional leaders in eliminating hunger in all its forms. This is in consideration of the country’s remarkable resource endowment, whereby 58% of its 75million ha of arable land had high potential for agricultural production [6]. However, Zambia’s past economic performance was typified by poignant statistical figures, such as high unemployment rate, low per capita income, and malnutrition [7]. Efforts within Zambia towards the dissolution of the challenge of poverty and hunger were diverse. One approach was the macroeconomic perspective, where the country needed to accelerate its real Gross Domestic Product (GDP) growth rate, to outpace that of population growth [8]. This per capita income approach was theoretically plausible, but only to the extent that a higher GDP growth rate could help to expand Zambia’s budgetary fiscal space and financial allocations to all sectors, including agriculture [9–11]. However, this approach could be long term. With no guarantees of equitable distribution of national resources, as demonstrated by Zambia’s large Gini coefficient averaging 0.5 since the 1990s [12], this paper argued that in the short to medium term, Zambia’s innovativeness within sectoral policies and programs such as the FBDP could help to reduce the country’s share of global poverty and hunger. The broad outreach of the agricultural sector and its direct impact on economically marginalized rural populations was an important lever for fighting global poverty and hunger. This was true for Zambia, where rural poverty was estimated at 78.8%, according to the 2022 Living Conditions Monitoring Survey report, by Zambia Statistics Agency (ZamStats). Therefore, given Zambia’s over-hyped, yet underutilized agricultural potential, it remained to be demonstrated how this sector was being prepared to bring about a “green revolution”.

Zambia’s agricultural policy for the 2012–2030 period was anchored on three main pillars, namely; agricultural sustainability, agricultural productivity, and agricultural diversification. The pillar of sustainability was propped up by Climate Smart Agriculture and agro-ecology (permaculture) strategies, including irrigation development, plus support to the general agricultural extension system [13,14]. Further, the Farmer Input Support Program supported the pillar of productivity promotion by assuring smallholder access to fertilizer and certified seed. A parallel but smaller Food Security Pack program under Zambia’s Ministry of Community Development and Social Services also supported crop productivity for marginalized but viable farmers [15–17]. Productivity promotion programs were supplemented by the smallholder credit facilitation (e.g., the Sustainable Agriculture Finance Facility), and general land husbandry activities within MoA’s extension system [18]. Additionally, the National Agricultural Mechanization Strategy was recently developed to promote efficient farming operations and expanded crop areas even among the crofters. This was appropriate for the commercial enterprise development under the FBDP. Lastly, several donor-supported projects, had been handy in promoting Zambia’s crop diversification efforts to address high incidences of malnutrition, evidenced by a low dietary diversity score and high levels of stunting [19]. Such projects included Community-Based Smallholder Irrigation, Market Oriented Rice Development Program, and Scaling-Up Nutrition, by Japan International Cooperation Agency and the United States Agency for International Development respectively.

Now, Zambia’s farm blocks were mere geographic spaces, identified to host diverse commercial farming activities, in some sort of regional “multi-farm production zones”. The main objectives of the program were to achieve a radical expansion in agricultural production, in job creation and industrialization of rural areas. Successful implementation of the FBDP through the commercialization of rural “idle” lands was expected to expand regional commodity trading, and enhanced local livelihoods. In essence, the farm blocks could boost Zambia’s agricultural policy, by ensuring sustainability, productivity, and diversification.

Private sector investments were extremely important for the actualization of the FBDP objectives. Nevertheless, that called for installation of key infrastructure, such as roads and powerlines by the public sector. Lack of this, and other key factors elucidated by Middelberg et al. [20], such as market access, remained key challenges to the full development of farm blocks in Zambia. The overall appropriateness of the FBDP initiative, in terms of its theoretical objectives, promised to be a game changer in Zambia’s agricultural sector. To maximize on this growth prospect, each of Zambia’s 10 provinces was designated to host at least one farm block, measuring about 100000 ha. However, due to the usufruct land users’ lack of financial capital and technical skills in commercial farm development, the invitation to participate in Zambian farm blocks was open to local and international investors.

This paper used an agricultural growth-mediated poverty reduction framework, through rural labor markets, backward and forward linkages, and infrastructure development [21]. Despite such projections for the FBDP in Zambia, a meta-analysis of global research by Akalu et al. [22], had demonstrated mixed outcomes. Matenga and Hichaambwa [23] also found this kind of outcome, from a study of 3 different commercial farm models in Zambia.

Belair et al. [24] studied large–scale land deals in seven SSA countries, and concluded that failures existed, due to flawed land acquisition processes, which resulted in local opposition to such projects. Another reason for the failures was financial difficulties to actualize the proposed investments. Within southern Africa, Baumert et al. [25] analyzed the impact of small, medium and large-scale land investments in Mozambique. Results indicated that these investments had created localized land scarcity, especially from large-scale commercial farming. These researchers stated, “The benefits from wage labor and local investments did not compensate rural populations for lost access to land”. Yet, small and medium investments provided greater on-farm employment benefits and other opportunities along the value chains. Incomes for small and medium-scale agricultural producers grew, which led to greater spill over effects in the local economy. In Zambia, Salverda and Nkonde [26] found that land administration excesses, lack of finance and presence of local residents on commodified land, were formidable challenges to the commercial development of large-scale land deals.

On the other hand, studies by Nthabeleng et al. [27]; Nhlengetfwa and Mamba [28] indicated that large-scale agricultural investments in Lesotho had improved food security and the nutritional status of local families. Such investments contributed to the improvement of income sources, and income levels for locals respectively in Eswatini.

The mixed results presented an important lesson for the FBDP in Zambia, which was promoting industrial agriculture mainly through LSLAs, less the crofters. This paper adopted a cautionary stance, by riding on two views; one, which asserted that policy making in SSA was not supported by robust research agendas [29], and another which highlighted the policy paradox, where program implementation in Africa seemed to be misaligned with existing research outcomes and recommendations [30]. Despite that, effective FBDP implementation in Zambia needed to be backed by relevant research outputs. Unfortunately, robust research activity to inform the FBDP implementation strategy in Zambia was lacking. Apart from Middelberg et al., who highlighted the national level challenges for the actualization of public and private farm block investments, host community viewpoints and needs remained unknown. While Matenga and Hichaambwa had looked at a similar topic in Zambia posteriorly, their study area was atypical of an intentional, formal and preplanned farm block development. Therefore, a knowledge gap existed, in terms of the expectations, and type of livelihood interventions needed to support the development of local communities. This paper endeavored to close this gap by revealing community level FBDP fears and expectations [31]. This was important for avoidance of the pitfalls of top-down implementation approaches [32]. The paper also examined the household income factors, to adduce possible areas of intervention for the achievement of positive community impacts and economic participation.

Given this background, this paper’s research problem was that the views and needs of farm block host communities in Zambia were not known. Yet, interactive sessions with the farm block host community members over a period, had revealed their profound fears and expectations, concerning the FBDP implementation process. The residents had expressed compunctious views about their “generous but non-beneficial” surrender of land to the FBDP. Second, following one of the community proposals to have local people empowered and transformed into farm block commercial farmers, this paper endeavored to investigate the viability of that idea, by assessing the generic drivers of household incomes to start with.

Lack of such knowledge could exacerbate poverty in affected communities, through misguided interventions and failure to register positive impacts on the welfare of local communities. This paper asked three research questions as follows; 1) What were the critical grassroots issues in Zambia’s FBDP? 2) What were the drivers of household incomes in farm block project locales? 3) How could the farm block community concerns and drivers of household incomes be used to formulate responsive policies to achieve inclusive development? This paper’s contribution to the literature is important, because it reveals, for the first time, local community fears and expectations, including the household income drivers, which could act as the springboard for local livelihood transformations.

The rest of this paper is organized as follows; section two highlights the methods of data collection and data analysis for the farm block community concerns and household income drivers. Results and discussion of the research outcomes are presented in section three. Concluding remarks accompanied with policy recommendations are offered in section four.

## 2. Methods

This paper used a mixed methods approach. The first part was a qualitative analysis of verbal submissions collected between 13^th^ April and 13^th^ June 2023, during community engagement meetings of the scoping exercise for Luena Farm Block’s Environmental and Social Impact Assessment (ESIA). The submissions were used to expound salient community forebodings, vis-a-viz possible solutions according to community expectations. The second part showed quantitative methods for the estimation of household income drivers, using data obtained from the socioeconomic component of the ESIA baseline survey. These data were collected between 23^rd^ and 29^th^ September 2023 by MoA field staff who were recruited as enumerators.

### 2.1. Ethical considerations

It was imperative to observe human participant ethics in this study, in respect of international norms set out in the Declaration of Helsinki, and local research ethics committees in Zambia. Since data were collected for ESIA in 2023, this research was reviewed posteriorly by Zambia National Health Research Authority, in response to the 2025 ethical waiver application. A conditional waiver (No. NHREB001/7/8/2025) was granted. However, the conditions were framed prospectively, which became infeasible. To elucidate on the data collection process, it suffices to state that before commencement of the data collection exercise, a request for the Luena Farm Block community consent was made through the local traditional leadership, which comprised of 3 Chiefs. The traditional leaders’ verbal consent was granted after the objectives of the exercise were explained to each of them. Following locally established protocols for gaining entry into communities; courtesy calls on the traditional leaders were spearheaded by senior government officials and were witnessed by local community stewards.

Before commencement of data collection, the exercise was publicized via an advert placed in the Times of Zambia newspaper, and another on local community radio. This communication was cascaded down to household level via street megaphone announcements by MoA’s National Agriculture Information Services. Door to door information sharing was facilitated by village head-persons. During data collection, no child participants were allowed, except in special cases, where older children were authorized by their parents/guardians to represent them. Feedback to the community was provided via ESIA disclosure meetings held posteriorly in some of the data collection venues. As part of the data curation exercise, the datasets were anonymized to protect respondent identities, and to desensitize individual and community images. Therefore, this research met the basic ethical standards, whereby prior informed consent was obtained, officially witnessed and documented.

### 2.2. Study area

Luena Farm Block was located in Kawambwa ditrict of Luapula province in Zambia. This farm block lied between Longitudes 29.10 and 29.60° east of the Greenwich Meridian, and between Latitudes −9.63 and −10.18° south of the Equator. It occupied Kawambwa’s central plateau, and its total coverage was 185000 ha, where 100000 ha of that were designated for farm plots. The rest comprised of swamps, forest reserves, human settlements and buffer zones. This areal extent represented 23% of the total surface area of Kawambwa district. Luena Farm Block was chosen for this study because it was one of the two pioneer farm blocks in Zambia.

### 2.3. Qualitative methods for data collection and analysis of community views

This subsection explains the qualitative research methods, which were used to collect and analyze the data. Table 1 shows the methods of data collection from different stakeholders.

**Table 1:** Stakeholder Engagement and Method of Consultation for Lena Farm Block, Kawambwa, Zambia.

SN	Stakeholder	Method	Resp.	Percent
1	Host community members (the affected party)	Outdoor plenary	2982	90.80
2	Provincial and District Development Coordinating Committees (development agents)	Focus Group Discussions	168	5.12
3	Business association, civic leaders, differently able persons, faith-based organizations, farmer groups, non-governmental organizations, and youth groups (interest groups)	Focus Group Discussions	116	3.53
4	Traditional leaders (influential persons)	Key Informant Interviews	13	0.40
5	Senior government officials (advisors on government policy)	Key Informant Interviews	3	0.09
6	Academia (expert advisors)	Key Informant Interviews	2	0.06
	Total		3284	100

Note: Provincial and District Development Coordinating Committees comprised of public and private sector professionals. Column 4 titled Resp. = number of respondents. Traditional leaders=3 chiefs plus 10 sub chiefs.

Verbal submissions from the different stakeholders were transcribed by means of electronic recorders, and rapporteur-recorded notes. Therefore, to construct logical information from the various submissions, which comprised of personal experiences, collective views (by voting), professional and expert advice from the different stakeholders, thematic and content analysis methods were used [33,34]. These methods were backed by ethnographic experience and expert advice. The process involved sorting and categorizing the submissions into broad themes that conveyed similar messages, using MAXQDA software. The messages were condensed and repackaged into consumable information for policy makers, and practitioners.

Table 2 shows the main thematic areas and content threads derived after sorting and repackaging. The outcome details are presented in subsection 3.1.

**Table 2:** Scoping Exercise Submissions from Luena Farm Block Community Engagement Meetings, Kawambwa, Zambia.

SN	Theme	Main message
1	Reticent project reception	The FBDP is not beneficial yet
2	Fear of displacement and loss of land user rights	We shall become landless beggars
3	Job scarcity and job quality	Few poor jobs from the few investors
4	Environmental damage and climate change	We shall lose our livelihood support base
5	Social change, cultural erosion, and heritage loss	We shall lose our identity to imported cultures

Note: Table contents compiled after analysis of all submissions.

### 2.4. Methods for quantitative data collection and estimation of household income factors

Quantitative data were collected by simple random sampling method, using in-depth household interviews conducted verbally and interactively at the respondent’s doorstep. The data-capturing tool was a semi-structured questionnaire, which was uploaded to KoboToolbox application, and was accessible to the enumerators on their smart phones. Responses were recorded by selecting answers from multiple-choice options, and by typing into spaces, which were provided for the unstructured questions that required probing. Door stickers were used to avoid double counting.

Before presentation of the data characteristics in subsection 2.5, it was prudent to point out a few things about the data early, because this assisted to justify the variable of interest and choice of analytical methods. According to the ZamStats household income was one of the quantifiable and objective measures of poverty in Zambia. Other measures were household consumption expenditure, and asset accumulation. The alternative approach was respondent self-reported poverty, which was scored on an ordinal scale, such as “very poor”, “moderately poor”, “poor”, etc. Nevertheless, self-reported poverty was considered to be subjective. Of all the measures of household economic welfare elucidated here, only household income was captured during the ESIA socioeconomic baseline survey in this particular case. This was used as an outcome variable despite its temporal dynamism.

Using this dataset, this paper’s first assumption was that total monthly income (mainly from farming sources) was an important indicator of informal economy livelihood support at the household level. The household was this paper’s unit of analysis. Now, to keep respondent confidentiality on household income disclosure, the survey instrument provided optional income bands for respondents to choose from. However, the minimum and maximum values of the income bands were averaged during data curation, to create a single income figure for each household, which could be manipulated statistically. The cleaned dataset showed that the household with the least monthly income earned ZMW50.05 (US$1.92), while the earnest household got ZMW10000.00 (US$384.62). This range can be presented as 50≤ZMW≤10000 for the whole dataset.

#### 2.4.1. Ordinary Least Squares regression.

To determe the household income factors for the farm block host community, the a priori quantitative analysis choice method was Ordinary Least Squares (OLS) regression. This model is given by;


$$\begin{array}{c} Y_{i}=\beta_{\text{0}}+\sum_{i=\text{1}}^{j}\beta_{j}X_{j}+\varepsilon_{i}i=\text{1},\text{2},\text{3}\ldots j \end{array}$$ (1)

where, $Y_{i}$ is the outcome or dependent variable (household income in this case) for household *i*, $X_{j}$ is a vector of independent variables (socioeconomic factors) which influence $Y_{i}$. $\varepsilon_{i}$ represents the error term that is independently and identically distributed (***iid***), with a mean of zero and constant variance $\sigma^{\text{2}}$ (homoskedasticity). Lastly, $\beta_{\text{0}}$ and $\beta_{j}$ are the parameters to be estimated.

**OLS conceptual framework.** Given the dataset for this study, the OLS model was reformulated to accommodate household and environment factors, which were deemed to impact household income in the study area. The model was specified as follows;


$$\begin{array}{c} n(Inc)=\beta_{\text{0}}+\sum_{i=\text{1}}^{j}\beta_{j}{lnX}_{j}+\delta LTS+\sum_{i=\text{1}}^{m}\delta_{m}D_{m}+\varepsilon_{i}i=\text{1},\text{2},\text{3}\ldots j, \end{array}$$ (2)

where ln(Inc) denoted the natural log for monthly household income, ${lnX}_{j}$ was natural log for the vector of household factors. LTS was a dummy for land tenure security, which was a factor at household level. $D_{m}$ was denoted by a vector of environment dummy variables, while $\varepsilon_{i}$ was the error term. The actual model based on Eq. 2, for 5 independent variables and 8 dummies became;


$$\begin{array}{cc} n(meanmonthlyincome)= & \beta_{\text{0}}+\beta_{\text{1}}ln(ageofhouseholdhead)+\beta_{\text{2}}ln(educationofhouseholdhead)\\ & +\beta_{\text{3}}ln(houseoldsize)+\beta_{\text{4}}ln(periodofstay)+\beta_{\text{5}}ln(cultivatedarea)\\ & +\delta_{\text{1}}(landtenuresecurity)+\delta_{\text{2}}(communityassistance)\\ & +\delta_{\text{3}}(socialwelfareassistance)+\delta_{\text{4}}(schoolfacilitypresence)+\delta_{\text{5}}(healthfacilitypresence)\\ & +\delta_{\text{6}}(accessroadpesence)+{\delta_{\text{7}}(electricityaccess)+\delta }_{\text{8}}(wateraccess)+\varepsilon_{i} \end{array}$$ (3)

However, the underlying Maximum Likelihood Estimator assumptions for OLS regression are quite restrictive. For example, Casson & Farmer; Schmidt & Finan; Flatt & Jacobs [35–37] explained that the theoretical assumptions for OLS regression are not easily met, depending on the data quality. The first assumption required that there was linearity for all variables in a multi-variate regression model. Second, there should be homoskedasticity of the error variance. Third, each of the terms in the model should be normally distributed. Fourth, the relationship between variables in the linear model should be independent and additive. Failure to meet these conditions could lead to erroneous conclusions and misguided policy recommendations. However, the difficulty of ensuring that the data quality meets all these conditions in real world research settings is difficult to guarantee. Yet, OLS regression remained a basic econometric model that is intuitively applicable in many research situations.

#### 2.4.2. Censored regression.

Censored regression became necessary in this paper for three reasons. The first was to account for possible respondent under-declaration of monthly incomes due to lack of proper income flow records. The second reason was to ensure that household income factors were determined using adequate amounts for local livelihood support, and investment capability, given the assertion by the host community members that they could be empowered to become commercial farmers. The third reason was to ensure the robustness of statistical outcomes.

Truncated models such as Heckman [38], could not be used since the farm block development impacts on household income were not only determined by one’s choice to participate in development activities, but also by the incidence of being resident in the area, hence the environment dummies. The hybrid Cragg’s double hurdle regression, which analyzes data at two levels; first by one’s choice to participate in a program, and second, by participation intensity [39], could not be used due to lack of explicit information on these aspects.

Given that background, censored regression was preferred. Nevertheless, to establish a suitable censoring point, the minimum wage set by Zambia’s Ministry of Labor and Social Security (MLSS), and the rural average income established by the ZamStats were considered for lower censoring limits. The unofficial but popular Basic Needs Basket, determined by the Jesuit Center for Theological Reflection (JCTR-BNB), was the minimum amount needed for a family of five to survive in Zambia’s most expensive cities. In this paper, this figure was regarded as one that could assure rural livelihood comfort (upper limit).

#### 2.4.3. Tobit regression.

Now, to compare these censoring points, standard Tobit regression model became useful, where more robust models such as the one described in the next subsection could not work, due to the heavy censoring. Introduced by Tobin [40], Tobit estimation is given by;


$$\begin{array}{c} y_{i}^{*}=\beta_{\text{0}}+\sum_{m=\text{1}}^{m}\beta_{m}X_{i}+\varepsilon_{i}\varepsilon_{i}\sim IN\left({\text{0},\sigma }^{\text{2}}\right) \end{array}$$ (4)

where, $y_{i}^{*}$ is a latent (or unobserved) variable, representing the average monthly income for household *i*, $X_{i}$ represents a vector of explanatory variables (household and environmental characteristics) associated with household *i*, and $\varepsilon_{i}$ is an error term. $\beta_{\text{0}}$ and $\beta_{m}$ are the unknown parameters to be estimated. Using a type 1 (i.e., left-censored) Tobit regression model, the household income censorship threshold c, is given as follows;


$$\begin{array}{c} y_{i}=\left\{ \begin{array}{c} @ly_{i}^{*},\;ify_{i}^{*}\leq c\\ y_{i}^{*},\;ify_{i}^{*}>c \end{array}\right. \end{array}$$ (5)

where, $y_{i}^{*}=\text{0},ify_{i}^{*}\leq c$, which represents the latent variable censored from the left when the value of $y_{i}^{*}$ is less or equal to c. On the other hand, $y_{i}^{*}=\text{1},\;ify_{i}^{*}>c$ denotes the continuous dependent variable in the observation range greater than c.

**Conceptual framework for Tobit regression.** Given the range for household monthly incomes: 50≤ZMW≤10000, the model was left-censored at the MLSS minimum wage of ZMW2861.36 (US$110.05); ZamStats rural average income of ZMW2112.20 (US$81.24); and at an arbitrary figure of ZMW250.00 (See reason in subsection 3.2). The JCTR-BNB of ZMW10800.56 (US$415.41) was the upper limit. The input and output variables were transformed into natural logarithms, except for the dummies. The recalibrated censorship thresholds, using log equivalents were as follows;


$$\begin{array}{c} y_{i}=\left\{ \begin{array}{c} @l\text{0},\;ify_{i}^{*}\leq \text{7}.\text{9}\text{5}\text{9}|\text{7}.\text{6}\text{5}\text{5}|\text{5}.\text{5}\text{2}\text{1}\\ \text{1},\;ify_{i}^{*}>\text{7}.\text{9}\text{5}\text{9}|\text{7}.\text{6}\text{5}\text{5}|\text{5}.\text{5}\text{2}\text{1} \end{array}\right. \end{array}$$ (6)

Now, since the study area’s maximum income did not exceed the JCTR-BNB figure, the right censoring threshold was equivalent to infinity. The upper limit became redundant, which justified the appropriateness of a left-censored model. Nevertheless, standard Tobit regression model is not robust to heteroskedasticity and non-normality of variance of the error term. Hence, Tobit regression can produce biased regression outputs [41].

#### 2.4.4. Censored least absolute deviations.

Finally, to determine the household income factors, Censored Least Absolute Deviations (CLAD) method introduced by Powell [42] was adopted. This robust semi-parametric model circumvents the regression outcome biases due to outliers, heteroskedasticity and non-normality of the error variance suffered in parametric analysis. This is achieved by minimizing the absolute errors from a selected data distributional quantile (percentiles, deciles, quintiles etc.), such as the median, 10^th^ or 90^th^ decile where skewness exists. CLAD is estimated by:


$$\begin{array}{c} {\hat{\beta }}_{CLAD}={min}_{\beta }\frac{\text{1}}{N}\sum_{I=\text{1}}^{N}\left|y_{i}-max\left(x_{i}^{\prime },\beta ,\text{0}\right)\right| \end{array}$$ (7)

where, N is the sample size, $y_{i}$ is the dependent variable, max(·) is a function of the predicted outcome, where $x_{i}^{\prime }$ is a vector of independent variables, and β is a vector of unknown coefficients to be estimated. 0 is the deviation from a given quantile. Else, Symmetrically Censored Least Squares (SCLS) also introduced by Powell is one of the usual alternatives.

### 2.5. Data characteristics

The ESIA socioeconomic survey data were captured from a sample of 5286 observations in Luena Farm Block in Luapula province, Zambia. Since the data were collected for a different purpose that did not demand statistical rigor, determination of the sample size was resource-based. However, the population of the study area was 39969, according to an estimate based on the 2022 census of population and housing data by ZamStats. Hence, the sample represented 13.23% of that population. The margin of error was ±1.26%, obtained by inclusion of a finite population correction factor (third term on the right hand side of Eq. 8);


$$\begin{array}{c} E=Z\times \sqrt{\frac{p(\text{1}-p)}{n}}\times \sqrt{\frac{N-n}{N-\text{1}}} \end{array}$$ (8)

where, E is the margin of error; Z = 1.96 (at 95% confidence level); and p = 0.5 denotes maximum variability. N = population size; and n = sample size. The low margin of error (i.e., < 5%) meant that this sample could represent the study area population with high precision [43].

Table 3 shows summary statistics for the socioeconomic data (S1 File). It was assumed that household variables were those with inherent influence on household income earning capabilities, especially those for the household head, who was the key decision-maker [44,45].

**Table 3:** Summary Statistics of Survey Data from Luena Farm Block, Kawambwa, Zambia.

VARIABLE	Mean	Std. Dev.	Min	Max
Outcome variable				
Mean monthly income (ZMW)	638.153	1360.839	50.05	10000.00
Household characteristics				
Age of household head (years)	43.078	15.704	14.00	110.00
Education of household head (years)	5.192	3.135	0.00	14.50
Household size (no.)	5.864	2.531	1.00	17.00
Period of stay in farm block area (years)	17.033	14.864	2.54	57.50
Cultivated area (ha)	1.438	2.148	0.00	80.00
Land tenure status (title deed certificate = 1)	0.055	0.229	0	1
Environment variables				
Community assistance (yes = 1)	0.647	0.478	0	1
Social welfare assistance (yes = 1)	0.153	0.360	0	1
School facility presence (yes = 1)	0.849	0.358	0	1
Health facility presence (yes = 1)	0.502	0.500	0	1
Access road presence (yes = 1)	0.705	0.456	0	1
Electricity access (yes = 1)	0.054	0.227	0	1
Water access (yes = 1)	0.253	0.435	0	1

No. of observations = 5286. ZMW = Zambian Kwacha (currency). Data sourced from ESIA baseline survey.

Operational environment related dummy variables were included because they determined the availability of opportunities for household income generation in the community [46].

## 3. Results and discussion

Detailed results for the two-part analyses of this paper are presented in this section. For information flow on the main research outcomes, results from quantitative models followed those from qualitative data analysis.

### 3.1. Community perspectives and concerns

To provide an answer to the first research question, this part presents qualitative data analysis results, using the ESIA scoping exercise submissions from Luena Farm Block. While Middelberg et al. highlighted Zambia’s national level FBDP issues, this paper’s annotation to this discourse was focused on the community concerns. Overall, the ESIA multi-stakeholder engagements established that 80% of the local communities were desirous of a fully developed farm block. However, these expressions were accompanied with concerns for the attendant negative environmental and social changes.

#### 3.1.1. Reticent project reception.

Zambia’s dual land tenure system and the hierarchical land administration structure presented a complex system that must be followed for a successful land alienation process [47]. Even though the Zambian government had the final authority in this process, the role of traditional leaders in land administration could not be overemphasized. In fact, even in the case of LSLAs orchestrated by the state, such as those meant for the FBDP, the local chiefs’ authorization, in a bottom up process was an important first step. Following that, some communities, through their traditional leadership in Zambia, were not receptive to the FBDP idea, mainly due to fears of LSLA-induced displacements [48,49]. To overcome that, community sensitizations on government intentions to transform a given portion of land into a farm block went alongside land negotiations between government and the local traditional leaders. However, sensitization activities could also end up raising difficult-to-fulfill promises, due to overtly positive messaging done with the intention of winning community cooperation.

Without the traditional leaders’ consent, state actors may not proceed to establish a farm block in a given area. It follows therefore, that where a community expressed dissatisfaction with the promise of real benefits, the setting up of a farm block in such an area could become jeopardized. In Zambia’s Eastern province for instance, the identification process for farm block land had to be restarted, after a turn down in Lundazi district. Traditional leaders in other districts had to be engaged. In another example, negotiations for the FBDP land had taken more than 20 years in some provinces of Zambia. However, in the case of Luena and Nansanga Farm Blocks in Zambia’s Luapula and Central provinces respectively, assurances of accelerated rural development and new job opportunities through infrastructure and commercial farm establishments assisted in securing land for the FBDP. However, the present misgivings were due to perceptions of missed employment and business opportunities. In Zambia’s Luena Farm Block, 47% of the respondents held views which supported renewed claims to their “ancestral” land [50,51]. With this background, land use activities which were conceptually inimical to the FBDP, such as artisanal mining and rosewood tree harvesting (*Pterocarpus chrysothrix* and *P. tinctorius*) had become rampant because they seemed to produce immediate benefits [52,53]. The relatively quick profits made from extractives were a major incentive to the proliferation of “legal and illegal” mining and logging outfits. It was argued that after all, the fringe benefits from these activities may never be enjoyed under the “formal” FBDP, which may have in fact, caused the community to lose doubly by its failure to empower locals, and by hindering windfall gains from alternative economic activities. However, 80% of the respondents pointed out that logging and artisanal mining were not economically helpful to the broader community. Meantime the Zambian government reserved superior sub-surface land use rights (e.g., granting of mining permits) [54] whose local community benefaction was being questioned, as the activities led to land degradation.

#### 3.1.2. Fear of displacement and loss of land user rights.

Since the FBDP promised to add a combined land size of more than 1000000 ha to the country’s commercial farm subsector, one could reasonably expect this land size not to be free of inhabitants. Most local communities comprised centuries-old villages, while new ones continued to spring up due to population growth. Although Zambia still had a low population density of around 28 persons/km^2^, with low and high extremes between rural and urban areas respectively, the prospect of creating Internally Displaced Persons due to FBDP LSLAs was almost certain [55]. In this particular study area, the fear of displacement was the most prominent issue for 90% the respondents. However, since the FBDP land allocation guidelines promoted the integration of commercial farms with pre-existing villages, the development of a Grievance Redress Mechanism (GRMs) and a Resettlement Action Plan (RAP) was initially overlooked, as formal backup systems for amicable resolution of disputes, and speedy restoration of livelihoods in case of displacements. Hence, the conflict resolution discourse in the FBDP remained ad-hoc. However, the availability of a GRM and a RAP could help the country avoid repeating the mistakes made in the 1950s, such as the Rhodesian (Zambia & Zimbabwe) Kariba dam construction displacements, and the protracted compensation processes that followed [56]. Under the FBDP, 56% of the affected parties thought that political channels were the most viable means of conflict resolution, while anti-land capture legal litigation by usufruct land users remained untenable. However, 75% of respondents indicated that there was little disquiet in Zambian farm block areas regarding displacement related conflicts, due to investor drought, despite large portions of land having been allocated on paper. Hence, simmering land conflicts between local communities and investors could be expected to boil over once the new landowners began to settle down. Otherwise, 70% of the local people called for their own empowerment and conversion into commercial farmers, as a safeguard measure against the creation of landless drifters.

#### 3.1.3. Job scarcity and quality.

The prospect of bringing about hundreds of diverse job opportunities to Zambia’s rural areas was one of the major selling points for the FBDP. However, this was proving to be a fleeting and fallacious panorama. Expressions of despair in terms of the small number of employment opportunities created thus far, was worsened by allegations of poor job quality. The seasonal, non-pensionable, labor-based contracts for menial tasks did not seem to satisfy a section of the youth population that expected skilled labor employment opportunities and respectable wages [57,58]. The few startup commercial farms in Zambia’s farm blocks lacked capacity to absorb the large number of trained job seekers, especially where physical labor endurance was preferable to soft skill qualifications. This explained the consternations of a significant portion of the youth group in Luena Farm Block. Worse still, some companies had previously tended to announce grandiose plans for their investment in Zambian farm blocks. For example, following the official commissioning of a private cassava (*Manihot esculenta*) production farm and biofuel blending plant in 2017, a 20000-strong out-grower backup in Luena Farm Block and surrounding areas was announced in 2018. This raised hopes for direct and indirect employment opportunities. However, nearly a decade later none of these aspects had come to fruition. This is an example of community over-priming, which raised local people’s expectations, but ended up contributing to the current youth consternations in terms of job scarcity. The lack of high voltage electric power for heavy industrial applications in Luena Farm Block area and the province at large, seemed to be a major limiting factor to the setting up of secondary level industries that could lead to the creation of more jobs in input supply, production, processing, and product distribution.

#### 3.1.4. Environmental damage and climate change.

The development of Luena Farm Block in Zambia’s Luapula province was locally concerning in terms of expected environmental changes. The local people’s concerns were related to how these radical environmental changes could impact their own livelihoods. Ecological transformations due to infrastructure and commercial farm developments would typically involve extensive vegetation clearing, leading to the loss of biodiversity [59,60]. The dense woodlands, which were a valuable source of forest products such as timber, fuelwood, wild fruits, honey, mushroom and herbal medicine, were part of the world’s forest cover, which also acted as carbon sinks and the earth’s lungs for gaseous exchange [61,62]. Forests also provided other ecological services, as flora and fauna habitats, climate buffers, and water catchment zones, which gave rise to many clear-water-carrying streams, in the case of Luena Farm Block. Land clearing was expected to cause the drying up of those streams, deepening of water tables, pollution and eutrophication of surface water bodies, as humans and animals got squeezed into few designated places [63–66]. Nostalgic prognoses for the imminent loss of that environment people were accustomed to, were echoed by 75% of respondents across the area. From this premise, those voices called for the Zambian government to ensure that investors effected proper mitigation measures, following the Zambia Environmental Management Agency (ZEMA) guidelines. However, investor compliance breaches, as demonstrated in Zambia’s mining sector, where chemical pollution occurred due to dam failure spillages, had highlighted how well intended projects could destroy local environments and livelihood sources [67]. While bemoaning the imminent changes, over 60% of local people called for the institutional strengthening of ZEMA, including MoA’s Department of Plant Quarantine and Phytosanitary Services, to ensure that strict standards were followed in the enforcement of biosafety regulations, to avoid the introduction of invasive species in terrestrial and aquatic environments.

#### 3.1.5. Social change, cultural erosion, and heritage desecration.

As almost 70% of the local people in Zambia’s Luena Farm Block welcomed investor, contractor, jobseeker, and mercantilist immigration, some of the serious community concerns were those related to cultural dilution, through heightened cross-cultural interactions. Over 57% of the local community showed conservative attitudes towards some contemporary social behaviors and practices. Preservation of local traditions, and customs in the Luena Farm Block area was called for [68,69]. On the other hand, extensive land clearing activities were feared to have the potential of uprooting important cultural heritage artefacts, including the removal of graveyards and shrines, leading to the desecration of the local community’s ancestral roots. It was deduced from expert opinion that these activities could go against the United Nations Declaration on the Rights of Indigenous Peoples (UNDRIP), and other agreements on the minority groups’ right to self-determination and cultural expression [70,71]. Additionally, it was believed that the resultant local population boom due to immigration could cause a disproportionate rise in the area’s crime rate and new diseases [72,73]. Because of the potential threats to the local culture, community proposed mitigation measures included; 1) strengthening the traditional leaders’ cultural custodianship and promotion of little-known traditional ceremonies, such as *Ituna-Imbusa* and *Chishinga*-*Malaila,* through Ministry of Local Government and Rural Development (MLGRD) to promote placeness or area identity, and tourism. 2) Creation of historical museums where cultural artefacts could be kept, and festivals of artistic and ceremonial nature, such as music and food fares, traditional games, folklore telling, writing and painting competitions could take place [74]. 3) Any rise in crime rate and the disease burden could be handled through enhanced police presence, religious moral teachings, and expanded health delivery systems respectively.

Considering the first research question, sub-section 3.1 provided the answer by bringing out some important insights into the pertinent FBDP host community forebodings in Zambia. While host communities acknowledged that environmental damage and social change were part of the price to pay for physical, economic, and social development, they contended that shared knowledge of what might be considered as “trivia” in the FBDP implementation processes was important for honing related policy measures for the improvement of household and community welfare in farm block host communities.

### 3.2. Household income factors

In the next half of this paper, focus shifted to the measurement of household income factors in Zambia’s Luena Farm Block, to answer the second research question. Table 4 shows the heteroskedasticity robust OLS output (S2 File), estimated using Stata-15. The model construction followed the conceptual model specified in Eq. 2, and expanded in Eq. 3.

**Table 4:** OLS Regression Results for Household Income Factors in Luena Farm Block, Kawambwa, Zambia.

VARIABLES	lnMean monthly income
Constant	5.400***
	(0.211)
lnAge of household head (years)	−0.104*
	(0.058)
lnEducation of household head (years)	0.118***
	(0.015)
lnHousehold size (no.)	0.074**
	(0.034)
lnPeriod of stay in farm block area (years)	0.030
	(0.019)
lnCultivated area (ha)	0.343***
	(0.021)
Land tenure (title deed certificate = 1)	0.711***
	(0.074)
Community assistance (yes = 1)	−0.115***
	(0.037)
Social welfare assistance (yes = 1)	0.131***
	(0.048)
School facility presence (yes = 1)	0.157***
	(0.051)
Health facility presence (yes = 1)	0.218***
	(0.038)
Access road presence (yes = 1)	−0.055
	(0.037)
Electricity access (yes = 1)	0.294***
	(0.075)
Water access (yes = 1)	0.031
	(0.040)
Observations	5286
R-squared	0.105

Standard errors in parentheses. *** p < 0.01, ** p < 0.05, * p < 0.1. Contents estimated from ESIA baseline data.

The OLS model diagnostics showed that multicollinearity was ignorable, since the mean variance inflation factor = 1.18, (i.e., less than 10 as a rule of thumb) [75]. This indicated that the model variables were independent and additive. Assuming linearity of independent variables, the Breusch-Pagan test result showed that heteroskedasticity was not significant; p > X^2^ = 0.324; indicating constant variance. However, without assuming linearity, the result of the White test indicated non-constant variance (i.e., presence of heteroskedasticity (p = 0.000)). The result of the normality test showed that the error variance was skewed (p > X^2^ = 0.000). Overall, the model coefficients should be taken as associations, because of the low R-squared value (0.105), which implied that only 10.5% of the variance in household income values was explained by the independent variables [76,77]. These diagnostic test results suggested that OLS could not be relied upon for the revelation of unbiased household income factors due to violations of some model assumptions.

Therefore, because of the reasons advanced in subsection 2.4.2., in respect of inexact household income disclosures, including uncertainty about the adequacy of the incomes for household livelihood support, Tobit models were introduced to assess the performance of three censoring yardsticks. Table 5 shows the Tobit model estimation results (S2 File) from Stata-15. It was found out that use of the MLSS minimum wage and ZamStats rural average income as thresholds led to the censoring of 5029 of 5286 (95%) observations respectively. This implied that 95% of the households in Luena Farm Block earned incomes, which were below Zambia’s official minimum wage and below the rural average income. This outcome was an important indicator of the community’s level of vulnerability.

**Table 5:** Tobit Estimation Results for Household Income Factors in Luena Farm Block, Kawambwa, Zambia, Using Different Lower Limits.

	lnMean monthly income		
VARIABLES	MLSS min. wage	ZamStats rural av.inc.	Arbitrary threshold
Constant	5.454***	4.097***	5.754***
	(0.480)	(0.699)	(0.176)
lnAge of household head (years)	−0.036	−0.068	−0.089*
	(0.129)	(0.188)	(0.049)
lnEducation of household head (years)	0.301***	0.420***	0.102***
	(0.050)	(0.072)	(0.013)
lnHousehold size (no.)	−0.116	−0.165	0.052*
	(0.075)	(0.110)	(0.029)
lnPeriod of stay in farm block area (years)	0.029	0.046	0.025
	(0.043)	(0.062)	(0.016)
lnCultivated area (ha)	0.221***	0.339***	0.277***
	(0.045)	(0.065)	(0.017)
Land tenure (title deed certificate = 1)	0.733***	1.030***	0.555***
	(0.132)	(0.194)	(0.059)
Community assistance (yes = 1)	0.190**	0.257**	−0.065**
	(0.082)	(0.120)	(0.030)
Social welfare assistance (yes = 1)	0.217**	0.331**	0.101**
	(0.099)	(0.144)	(0.040)
School facility presence (yes = 1)	0.005	0.003	0.073*
	(0.115)	(0.167)	(0.043)
Health facility presence (yes = 1)	0.229***	0.346***	0.194***
	(0.082)	(0.120)	(0.031)
Access roads presence (yes = 1)	−0.167**	−0.239**	−0.071**
	(0.080)	(0.116)	(0.031)
Electricity access (yes = 1)	−0.250	−0.376	0.168***
	(0.168)	(0.246)	(0.061)
Water access (yes = 1)	0.203**	0.265**	0.058*
	(0.082)	(0.120)	(0.033)
var (e.lnHousehold income)	1.478***	3.153***	0.911***
	(0.162)	(0.353)	(0.022)
Log-likelihood	−1058.100	−1162.732	−6296.025
Log-likelihood ratio	134.59	131.42	550.18
Observations	5286	5286	5286

Standard errors in parentheses. *** p < 0.01, ** p < 0.05, * p < 0.1. Contents estimated from ESIA baseline data.

However, to uphold the realism of the area’s socioeconomic situation, the censorship threshold was lowered to an arbitrary figure of ZMW250.00 ($9.62) (see mean of monthly incomes in Table 3). This helped to reduce on the number of censored observations. Using this figure, only 1577 of 5286 (30%) observations were censored. Now, results in columns 2 and 3 could not be obtained using CLAD or SCLS, due to the heavy censoring of data.

The Tobit model in column 4 of Table 5 demonstrated a better (i.e., higher value of the) log-likelihood ratio, which implied that the addition of one more independent variable (i.e., socioeconomic factors) improved the explanatory power of the model [78], as opposed to the nested model (with constant only (degree of freedom = 13)). Thus, the best censoring threshold in this case, was none of the officially established poverty datum lines in Zambia.

Finally, Table 6 shows results from the CLAD model (S2 File) as explained in subsection 2.4.4, using the lower censoring point established arbitrarily, given the average monthly income indicated in Table 3. These results provided the answer to the second research question about which factors affected household incomes in farm block host communities.

**Table 6:** CLAD Estimation Results for Household Income Factors in Luena Farm Block, Kawambwa, Zambia.

VARIABLES	lnMean monthly income
Constant	7.542***
	(0.322)
lnAge of household head (years)	−0.220**
	(0.086)
lnEducation of household head (years)	0.113***
	(0.027)
lnHousehold size (no.)	0.145**
	(0.056)
lnPeriod of stay in farm block area (years)	−0.018
	(0.029)
lnCultivated area (ha)	0.357***
	(0.036)
Land tenure (title deed certificate = 1)	1.643***
	(0.118)
Community assistance (yes = 1)	0.009
	(0.057)
Social welfare assistance (yes = 1)	0.051
	(0.073)
School facility presence (yes = 1)	−0.057
	(0.077)
Health facility presence (yes = 1)	0.240***
	(0.062)
Access roads presence (yes = 1)	−0.280***
	(0.060)
Electricity access (yes = 1)	0.106
	(0.116)
Water access (yes = 1)	0.154**
	(0.064)
Observations	5284

Bootstrap standard errors in parentheses. *** p < 0.01, ** p < 0.05, * p < 0.1. Estimated from ESIA baseline data.

Estimation results showed that a 1-percentage point rise in education of the household head was positively associated with a 1.3-percentage point rise in household income (model coefficient for education). The same logic followed for household size, and cultivated area. Possession of TDCs to signify land tenure security was associated with raised average household incomes. Environment dummies that were positively associated with higher average incomes were health facility presence, and water access. Otherwise, age, and access roads were negatively associated with household incomes. The alternative SCLS model yielded results (S2 File) that were similar to those from the OLS model (See Table 7 in S1 Appendix).

Interpretation of these results was as follows: Education of the household head (who approved most household decisions in this cultural context) could positively contribute to household income due to heightened literacy level, and enhanced household capacity to deal with formal processes and technical information. This outcome agrees with findings by Kelechi et al. [79]. A larger household size implied that more labor units were available to support larger scale and diverse household economic activities. The pooling of incomes from diversified sources resulted in better household income. Mbewana and Kaseeram [80] alluded to this effect. The larger the cultivated area, the larger the output volumes, and household income from the sale of surplus produce [81]. Furthermore, it was long established in the literature, that possession of a TDC meant having land tenure security, which was associated with the ability to obtain credit for fixed investments in land, ultimately leading to higher household incomes. However, the ability to obtain a state-issued TDC could in fact, signify prior higher earning capacity or a steady average household income flow. This could be by wage earners, since most usufructs failed to do so, as they encountered prohibitive costs in a usually bureaucratic TDC acquisition process. Byamugisha [82]; Sitko et al. [83] ably elaborated this effect.

In terms of the influence of environmental factors on household income, only health facility presence, and water access were positively associated with higher household incomes. Health facility presence was associated with higher household incomes through improved productivity, due to the so-called health capital, as indicated by Abdi Ali et al. [84]. This outcome seemed to validate the “health is wealth” mantra in public health parlance. Finally, this model demonstrated that water access was another important factor associated with household incomes. In as much as water was a necessity for life support, it was also an important economic commodity, because it supported human economic activity, including off-season agricultural production. Further, water access improved community hygiene, thereby reducing the overall disease occurrence. Magbonde et al. [85] highlighted this relationship.

Meanwhile, the negative association of age of the household head with household incomes meant that where household heads were older, the ability to raise income had diminished. Zimmer and Das [86] had established this situation in their study of SSA. While the presence of access roads provided a vital economic link for rural areas, the same roads could facilitate the silent exodus of the economically active age groups from many households, as they sought better opportunities elsewhere, leaving behind women and the elderly. Vercillo and Frayne [87] elaborated this phenomenon.

## 4. Conclusion

The FBDP in Zambia was launched for the promotion of agricultural commercialization by utilizing the country’s rural arable lands. However, negative changes due to industrial agriculture developments could be radical in scope and scale. This paper highlighted the undocumented concerns and needs of the host communities. Addressing them could assure the FBDP implementation success. Based on community-proposed solutions, a partial answer to the third research question was provided, by recommending that; 1) the FBDP should ensure through MLGRD that the local traditional leaders are streamlined in the farm block development process to protect local community interests. 2) In partnership with MLSS, and Department of Resettlement, the FBDP should devise GRMs and RAPs, to protect local people from falling into landless destitution due to LSLA displacements. 3) The FBDP should ensure timely actualization of investment pledges by new landowners, to reduce job scarcity in farm block areas. 4) ZEMA and MoA should ensure environmental protection in farm blocks by countering the commercial farm monocultures, with establishment of new forest reserves to assure ecosystem diversity, climate resilience, and usufruct livelihood support. 5) The traditional leaders’ cultural custodial role should be assured through MLGRD, by the promotion of traditional ceremonies, and the creation of local museums to host regular cultural events.

The second part of this paper revealed that there were high levels of economic vulnerability in farm block host communities, whereby household incomes where below Zambia’s official minimum wage. However, several household and environment factors affected household incomes positively. Those factors formed the basis on which host communities could grow their household incomes to become active economic participants in the FBDP.

From these findings, this paper completed the answer to the third research question, which sought to establish how the drivers of household incomes could be used to formulate policies that responded to the social and economic developments in farm block areas in Zambia. The paper recommended that; 1) Education should be promoted in farm block areas through the Ministry of Education in Zambia, by building more schools, sustain teacher recruitment and retention programs, including youth skills training centers for practical applications. 2) Mechanization should be promoted through MoA and private players, to achieve cultivated area expansion and production efficiency. 3) Land tenure security should be promoted through the Ministry of Lands and its sub-regional agencies, by pursuing a special program to offer individual and block TDCs to deserving farm block dwellers. 4) Health services delivery should be strengthened through the Ministry of Health in Zambia, to ensure that the local populations fully participated in emerging economic activities. 5) Modern utility services (e.g., water utility companies) should be introduced through responsible ministries, such as Ministry of Water Development and Sanitation, to promote higher household incomes through provision of a life-supporting and economic commodity like safe water. 6) Industrialization of farm blocks should be accelerated through Zambia Development Agency, to stem the exodus of youths from rural host communities.

Finally, when seen from the lens of a microeconomic theoretical framework which assumed that household poverty reduction could be mediated through agricultural commercialization, this scholarly inquiry into the viewpoints and needs of farm block host communities highlighted a number of sticky points to this claim. Therefore, this paper urged caution in the implementation of the FBDP, to ensure sustainable development. An overall improvement in these aspects could foster broader gains and fairer rural livelihoods. This could help to prepare the usufructs’ eventual transformation into local commercial farmers, and the reversal of the negative narratives about Zambia’s FBDP. Macroeconomic level outcomes could include a higher GDP, a lower Gini coefficient and lower malnutrition statistics. This could signify a reduction in rural poverty and better GHI rankings for Zambia, which could be an important contribution towards the same in SSA, and towards the achievement of UNSDGs 1 and 2.

However, the limits of this research were four-fold. First, the quantitative part of this paper was a causal inference research and not an impact evaluation since there was no control group to assist the evaluation of treatment effects. Second, household size was inclusive of children and the elderly who could not take part in household productive activities. This could distort the model outcomes. Third, the socioeconomic data were limited to one outcome variable (household income) as a livelihood gauge. Fourth, the data were for a singular province and were cross-sectional, having been collected for ESIA purposes. Thus, future research on Zambian farm blocks should consider program impact evaluation. Second, future research should consider using clear-cut data, which depicts actual household economic agents. Third, future research should ponder using variables that constituted holistic household welfare systems in farm block areas. Fourth, future research should consider using longitudinal data to control for regional disparities, including temporal variations.

## Supporting information

S1 File Excel datafile. (XLSX)

S2 File Stata Do-file. (XLSX)

S1 Appendix Table 7. SCLS Estimation Results for Drivers of Household Incomes in Luena Farm Block, Kawambwa, Zambia. (DOCX)
