# Supplementary material for: Community perspectives on the Zambian Farm Block Development Program and an evaluation of household income factors: Lessons from Kawambwa district
Source: PLoS One. 2026 Aug 28;21(8):e0356479. doi: 10.1371/journal.pone.0356479 (PMC13524339; doi:10.1371/journal.pone.0356479)
Supplement: Supplementary file 1 [file pone.0356479.s003.docx]

**Table 7. SCLS Estimation Results for Drivers of Household Incomes in Luena Farm Block, Kawambwa, Zambia.**

| VARIABLES | Mean monthly income |
| --- | --- |
| Constant | 5.400*** |
|  | (0.212) |
| lnAge of household head (years) | -0.104* |
|  | (0.058) |
| lnEducation of household head (years) | 0.118*** |
|  | (0.015) |
| lnHousehold size (no.) | 0.074** |
|  | (0.036) |
| lnPeriod of stay in farm block area (years) | 0.030 |
|  | (0.019) |
| lnCultivated area (ha) | 0.343*** |
|  | (0.022) |
| Land tenure (title deed certificate=1) | 0.711*** |
|  | (0.081) |
| Community assistance (yes=1) | -0.115*** |
|  | (0.035) |
| Social welfare assistance (yes=1) | 0.131*** |
|  | (0.048) |
| School facility presence (yes=1) | 0.157*** |
|  | (0.053) |
| Health facility presence (yes=1) | 0.218*** |
|  | (0.038) |
| Access roads presence (yes=1) | -0.055 |
|  | (0.037) |
| Electricity access (yes=1) | 0.294*** |
|  | (0.068) |
| Water access (yes=1) | 0.031 |
|  | (0.042) |
| Observations | 5286 |

Standard errors in parentheses. *** p<0.01, ** p<0.05, * p<0.1. Contents estimated from ESIA baseline data.
